# Galectin-3 (MAC-2) controls phagocytosis and macropinocytosis through intracellular and extracellular mechanisms

**DOI:** 10.3389/fncel.2022.949079

**Published:** 2022-10-05

**Authors:** Shlomo Rotshenker

**Affiliations:** Department of Medical Neurobiology, Institute for Medical Research Israel-Canada (IMRIC), Hebrew University Faculty of Medicine, Jerusalem, Israel

**Keywords:** Galectin-3 (Gal-3), microglia, macrophage, Schwann cell, astrocyte, phagocytosis, macropinocytosis, cytoskeleton

## Abstract

Galectin-3 (Gal-3; formally named MAC-2) is a β-galactoside-binding lectin. Various cell types produce Gal-3 under either normal conditions and/or pathological conditions. Gal-3 can be present in cells' nuclei and cytoplasm, secreted from producing cells, and associated with cells' plasma membranes. This review focuses on how Gal-3 controls phagocytosis and macropinocytosis. Intracellular and extracellular Gal-3 promotes the phagocytosis of phagocytic targets/cargo (e.g., tissue debris and apoptotic cells) in “professional phagocytes” (e.g., microglia and macrophages) and “non-professional phagocytes” (e.g., Schwann cells and astrocytes). Intracellularly, Gal-3 promotes phagocytosis by controlling the “eat me” signaling pathways that phagocytic receptors generate, directing the cytoskeleton to produce the mechanical forces that drive the structural changes on which phagocytosis depends, protrusion and then retraction of filopodia and lamellipodia as they, respectively, engulf and then internalize phagocytic targets. Extracellularly, Gal-3 promotes phagocytosis by functioning as an opsonin, linking phagocytic targets to phagocytic receptors, activating them to generate the “eat me” signaling pathways. Macropinocytosis is a non-selective endocytic mechanism that various cells use to internalize the bulk of extracellular fluid and included materials/cargo (e.g., dissolved nutrients, proteins, and pathogens). Extracellular and intracellular Gal-3 control macropinocytosis in some types of cancer. Phagocytosed and macropinocytosed targets/cargo that reach lysosomes for degradation may rupture lysosomal membranes. Damaged lysosomal membranes undergo either repair or removal by selective autophagy (i.e., lysophagy) that intracellular Gal-3 controls.

## Introduction

Galectin-3 (Gal-3) is a β-galactoside-binding protein, encoded by the Lgals3 gene (Barondes et al., 1994; Dumic et al., 2006; Yang et al., 2008; Johannes et al., 2018; Barake et al., 2020). Gal-3 was formally named MAC-2 when first identified as a cell surface protein on thioglycollate-elicited mouse macrophages (Ho and Springer, 1982). Of note, Gal-3 was previously known also as carbohydrate-binding protein (CBP)-35, IgEBP, CBP-30, and more. Many cell types produce Gal-3 under either normal and/or pathological conditions. Gal-3 presence in cells' nuclei and cytoplasm, secretion from producing cells, and association with cells' plasma membranes enable Gal-3 to be involved in diverse intracellular and extracellular functions (Dumic et al., 2006; Yang et al., 2008; Barake et al., 2020). The current review focuses on intracellular and extracellular roles that Gal-3 plays in controlling phagocytosis and macropinocytosis; phagocytosis in professional phagocytes (e.g., microglia and macrophages) and non-professional phagocytes (e.g., Schwann cells and astrocytes); and macropinocytosis in some types of cancer cells. Hence, studies relating Gal-3 to phagocytosis and macropinocytosis are discussed herein.

Phagocytosis is a receptor-mediated process, whereby phagocytes engulf and internalize phagocytic targets (≥0.5 μm) destined for degradation in lysosomes (e.g., apoptotic cells, tissue debris, pathogens, and malignant cells); thus, it is essential for homeostasis and combating infection and diseases. Phagocytic targets trigger phagocytosis by binding their cognate phagocytic receptors, activating them to generate the “eat me” signal transduction pathways, directing the cytoskeleton to produce the mechanical forces that drive the structural events of engulfment and internalization on which phagocytosis depends. First, surface membranes, in the form of filopodia and lamellipodia, protrude and engulf phagocytic targets, and then filopodia/lamellipodia retract to internalize the targets. I shall discuss in this context how Gal-3 promotes phagocytosis by advancing “eat me” signaling pathways in phagocytes. Phagocytic targets may bind phagocytic receptors directly and/or indirectly. Indirect binding depends on opsonins linking phagocytic targets to the phagocytic receptor by binding targets and complementary phagocytic receptors at the same time, activating phagocytic receptors to generate the “eat me” signal transduction pathways. I shall discuss in this context how Gal-3 functions as an opsonin. Notably, receptor-mediated phagocytosis is subjected to modulation between efficient and inefficient activation states. Thus, the presence of phagocytic receptors is not an indication that they mediate phagocytosis (discussed in an upcoming section).

Macropinocytosis is an evolutionary conserved non-selective endocytic mechanism that various cell types, from amoeba to mammalian malignant cells and macrophages, use for the uptake of extracellular fluid, including nutrients (e.g., amino acids and glucose), proteins (e.g., albumin and antigens), pathogens and debris (e.g., King and Kay, 2019; Palm, 2019; Kay, 2021). Macropinocytosis, such as phagocytosis, depends on the cytoskeleton, driving the production of plasma membrane ruffles/lamellipodia that first engulf and then internalize extracellular fluid and included cargo (e.g., Mylvaganam et al., 2021). However, distinct from phagocytosis, the activation of macropinocytosis does not depend on the interaction between cargo and cognate receptors. Extracellular and intracellular Gal-3 activate macropinocytosis in some types of cancer cells (discussed in an upcoming section).

Phagocytosed and macropinocytosed targets/cargo that reach lysosomes for degradation can rupture lysosomal membranes. The damaged endomembranes may undergo either repair or removal through selective autophagy named lysophagy. Intracellular Gal-3 controls both repair and lysophagy (Jia et al., 2020) (discussed in an upcoming section).

## Gal-3 promotes phagocytosis—first indications in Schwann cells, microglia, and macrophages

A series of earlier studies provided the first indications that Gal-3 controls phagocytosis in microglia and macrophages (i.e., professional phagocytes) and Schwann cells (i.e., a non-professional phagocyte) by showing that the upregulation of the Gal-3 expression is positively correlated with phagocytosis activation. Of note, some of the earlier studies named Gal-3, MAC-2. We used peripheral and central nervous system (PNS and CNS) nerve injury and experimental allergic encephalomyelitis (EAE), an animal model for multiple sclerosis (EAE), to study the *in vivo* clearance of myelin debris (also referred to as degenerated myelin). Myelin debris is the breakdown product of intact myelin, which is a specialized extension of the plasma membranes of Schwann cells in PNS and of oligodendrocytes in CNS. Intact myelin normally surrounds the larger diameter axons, enabling their function. Myelin breaks in EAE and following traumatic injury to PNS and CNS myelinated axons distal to lesion sites where, respectively, PNS and CNS Wallerian degeneration develop (e.g., reviewed in Rotshenker, 2011, 2015). In all instances, the clearance of this tissue debris is critical to repair.

### Schwann cells

We showed that Schwann cells neither displayed phagocytic activity nor expressed Gal-3 in intact PNS nerves, whereas, following traumatic injury, Schwann cells residing in PNS Wallerian degeneration upregulated the Gal-3 expression and further internalized myelin debris (Reichert et al., 1994). In this regard, Schwann cells mimicked monocyte-derived macrophages that are normally recruited in PNS Wallerian degeneration; the two cell types expressed Gal-3 and phagocytosed myelin debris. We also showed that exogenously applied lactose and galactose, which block Gal-3 binding β-galactoside (Frigeri et al., 1990), inhibited Schwann cells' ability to clear the debris in PNS nerve explants undergoing *in vitro* PNS Wallerian degeneration in the absence of recruited macrophages. These findings suggest that extracellular Gal-3 that Schwann cells produced and secreted promoted myelin debris phagocytosis through a lactose/galactose-inhibitable extracellular mechanism (discussed in detail in an upcoming section).

Granulocyte macrophage colony-stimulating factor (GM-CSF), which is a pro-inflammatory cytokine and a granulocyte and monocyte growth factor, contributed to the upregulation of the Gal-3 expression in PNS Wallerian degeneration. We showed (Saada et al., 1996; Be'eri et al., 1998) (a) that GM-CSF, which is mostly produced by fibroblasts in PNS Wallerian degeneration, upregulated the Gal-3 expression in Schwann cells and macrophages, and (b) that GM-CSF induced bone-marrow derived monocyte precursor cell that did not express Gal-3 to differentiate into Gal-3-expressing macrophages. These findings are in line with observations by Elliott et al. (1991) that macrophages in GM-CSF transgenic mice expressed increased levels of Gal-3.

### Microglia

We studied myelin debris phagocytosis and Gal-3 expression in microglia *in vivo* and *in vitro*: *in vivo*, in intact and injured optic nerves (Reichert and Rotshenker, 1996) and EAE (Reichert and Rotshenker, 1999), and *in vitro*, in cultured microglia derived from intact optic nerves (Reichert and Rotshenker, 1996). We showed that microglia in intact optic nerves neither displayed phagocytic activity nor expressed Gal-3 and that, distal to lesion sites, where CNS Wallerian degeneration developed following optic nerve injury, microglia failed to upregulate the Gal-3 expression or phagocytose myelin debris. We made similar observations studying intact and injured spinal cords. In contrast to the failure to upregulate the Gal-3 expression and phagocytosis in CNS Wallerian degeneration, microglia upregulated both the Gal-3 expression and phagocytosis of myelin debris *in vivo* in EAE (Reichert and Rotshenker, 1999). Furthermore, cultured microglia that migrated out from explants of intact normal optic nerves upregulated the Gal-3 expression and phagocytosis of myelin debris *in vitro* (Reichert and Rotshenker, 1996). Taken together, microglia upregulated the Gal-3 expression and phagocytic activity *in vivo* in EAE and *in vitro* in cultured microglia but not *in vivo* in CNS Wallerian degeneration. Thus, the upregulation of the Gal-3 expression and phagocytosis activation positively correlated with each other, suggesting that Gal-3 plays a role in promoting phagocytosis.

Importantly, optic nerve microglia that failed to phagocytose myelin debris in CNS Wallerian degeneration upregulated the expression of phagocytic receptors CR3 (complement receptor-3, formally named MAC-1) and SRAI/II (scavenger receptor AI/II) (Bell et al., 1994; Reichert and Rotshenker, 1996), efficiently mediating myelin debris phagocytosis in cultured primary microglia and macrophages (Reichert et al., 2001; Reichert and Rotshenker, 2003; Rotshenker, 2003). Thus, the *in vivo* presence of phagocytic receptors is not an indication that they mediate phagocytosis, suggesting that receptor-mediated phagocytosis is subjected to modulation between efficient and inefficient activation states.

Of note, in injured CNS, Gal-3 expression and phagocytosis were activated at injury sites where the physical impact has occurred but not distal to injury sites where CNS Wallerian degeneration developed (Reichert and Rotshenker, 1996). The two regions differ. The non-neuronal cell population in CNS Wallerian degeneration is composed of resident microglia, astrocytes, and oligodendrocytes. At CNS injury sites, where the physical impact had occurred, monocyte-derived macrophages from ruptured vasculature and potentially meningeal cells joined the resident non-neuronal cell population.

### Macrophages

Sano et al. (2003) studied FcγR (Fcγ receptor)-mediated phagocytosis of IgG-opsonized erythrocytes in cultured Gal-3-deficient and wild-type primary macrophages, showing that phagocytosis was delayed in Gal-3 deficient macrophages. Thus, the Gal-3 expression and phagocytosis positively correlated with each other in macrophages too. They further showed that exogenously applied lactose, which blocks Gal-3 binding β-galactoside, did not affect phagocytosis, suggesting that Gal-3 promoted phagocytosis through intracellular and not extracellular mechanisms. By contrast, Karlsson et al. (2009) suggested that Gal-3 promotes phagocytosis through extracellular and not intracellular mechanisms based on their findings that exogenously applied Gal-3 augmented lactose-inhibitable phagocytosis of apoptotic neutrophils in macrophages. The opposing conclusions reached by Sano et al. (2003) and Karlsson et al. (2009) can be reconciled by the fact that each group studied phagocytosis *via* a different phagocytic receptor. Sano et al. (2003) studied FcγR-mediated phagocytosis of IgG-opsonized erythrocytes. Karlsson et al. (2009) studied phagocytosis of apoptotic cells *via*, most probably, MerTK (a member of the Mer/Tyro3/Axl tyrosine kinase TAM receptor family) that Caberoy et al. (2012) showed mediated the phagocytosis of Gal-3-opsonized apoptotic cells (discussed in detail in an upcoming section). Moreover, subsequent studies showed that Gal-3 promotes phagocytosis by both intracellular and extracellular mechanisms: intracellular Gal-3 by controlling the “eat me” signal transduction pathways and extracellular Gal-3 by functioning as an opsonin (discussed in detail in upcoming sections).

## Intracellular Gal-3 promotes phagocytosis in microglia and macrophages by controlling “eat me” signal transduction pathways that target the cytoskeleton

### Phagocytic capacity and cell morphology correlate in microglia

In microglia, efficient phagocytosis is often correlated with “amoeboid” morphology and deficient phagocytosis with highly “branched/ramified” morphology, two extremes in a spectrum. Amoeboid microglia are usually round or oval-shaped, occasionally sending out pseudopodia of various lengths. Highly branched/ramified microglia send out very thin and very long branches that give rise to secondary and tertiary spiky daughter branches. The transition between the two phenotypes is through intermediate steps. Initially, amoeboid microglia extend short and thick processes that gradually become very thin, very long, and highly branched/ramified (Rio-Hortega, 1932; Cunningham et al., 2013). In the developing CNS, microglia display amoeboid morphology and efficient phagocytosis, enabling microglia to remove neurons and eliminate/strip/prune synapses, shaping thereby the CNS neuronal circuitry. Toward adulthood, microglia morphology transforms gradually from amoeboid to highly branched/ramified and phagocytosis subsides (Rio-Hortega, 1932; Sierra et al., 2016; Tay et al., 2016). Yet, microglia remain phagocytic active in specific regions of the adult CNS where neurons are continuously replaced, e.g., the hippocampus (Sierra et al., 2010) and olfactory bulb (Ribeiro Xavier et al., 2015). Microglia morphology reverts toward amoeboid and phagocytic activity resumes *in vivo* in disease and *in vitro* in culture (Rio-Hortega, 1932; Gitik et al., 2010, 2011; Reichert and Rotshenker, 2019). The molecular mechanisms that control microglia transformation from one phenotype to the other remain largely unknown. Gal-3 could affect both microglia morphology and phagocytosis by controlling signal transduction pathways that direct the cytoskeleton to generate the mechanical forces that drive both phagocytosis and changes in cell morphology (Reichert and Rotshenker, 2019) (discussed in detail below).

### The cytoskeleton generates the mechanical forces that drive phagocytosis

Protrusion and retraction of filopodia/lamellipodia are hallmarks of phagocytosis in professional phagocytes and cell movement and migration in normal non-phagocytic cells and metastatic cancer cells. As such, phagocytosis and cell movement/migration share many of the signaling molecules that control the protrusion and retraction of plasma membranes. Therefore, findings in phagocytes and findings in non-phagocytic cells that are relevant to phagocytosis are discussed herein.

**Figure 1:**
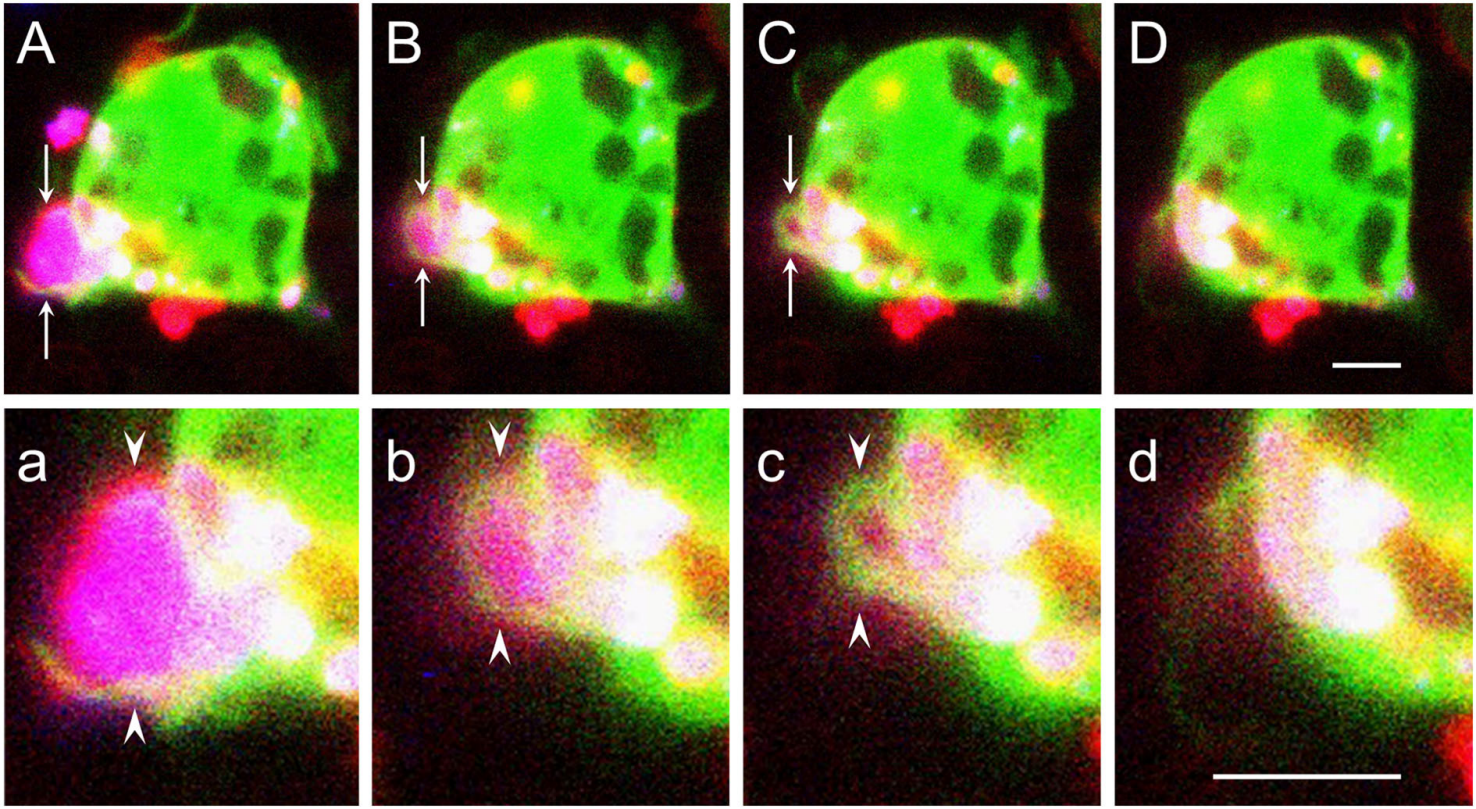
Filopodia/lamellipodia extend and engulf myelin debris and then retract and internalize the debris. [**(A–D)** and **(a–d)**] Time-lapse cinematography images of successive steps during live ongoing phagocytosis of a myelin debris particle by a wild-type macrophage. **(A–D)** Arrows mark filopodia/lamellipodia-like structures (green and/or red) that form a phagocytic cup that engulfs a myelin particle (purple), and **(a–d)** arrowheads mark enlarged images of the phagocytic cup. First **(A,a)**, filopodia/lamellipodia engulf myelin. Then, [**(B–D)** and **(b–d)**], filopodia/lamellipodia retract, pulling the debris into the phagocyte. Timing after the onset of phagocytosis in **(A,a)**, **(B,b)**, **(C,c)**, and **(D,d)** are, respectively, 3.5, 6.5, 7.5, and 8.5 min. Live ongoing phagocytosis was monitored continuously by confocal fluorescence microscopy at a single plane of focus. The macrophage cytoplasm is visualized by Ca+2 indicator Fura-4 (green), myelin by DiIc18 (blue), and macrophage cell membrane by RH237 (red). The fluorophore RH237 was also incorporated into myelin that turned purple (blue + red). Each image is a composite of three sequential scans taken during ongoing phagocytosis, one for each fluorophore, resulting in cytoplasmic Ca+2 indicator Fura-4 (green) obscuring the RH237 (red) labeled thin plasma membrane apart from in **(A,a)** where the red labeled plasma membrane is clearly seen. Bars: 5 μm for **(A–D)** in **(D)** and 5 μm for **(a–d)** in **(d)**. Figure modified after Hadas et al. (2012).

Using cultured primary microglia and macrophages, we previously showed that phagocytosis of myelin debris, which is mostly CR3/MAC-1 and to a lesser extent SRAI/II mediated in the absence of anti-myelin antibodies (Reichert et al., 2001; Reichert and Rotshenker, 2003; Rotshenker, 2003), involved two related structural events. Filopodia/lamellipodia protruded and engulfed myelin debris, and then, filopodia/lamellipodia retracted and internalized the debris (Hadas et al., 2012; Figure 1). Protrusion of filopodia/lamellipodia requires that filaments of actin (F-actin) undergo remodeling; i.e., existing F-actin disassemble and new F-actin assemble in a new configuration, forcing plasma membranes to protrude (Oser and Condeelis, 2009; Bernstein and Bamburg, 2010; Svitkina, 2018). Cofilin, an actin-depolymerizing factor, plays a key role in initiating F-actin remodeling by disassembling existing F-actin. Cofilin activity is subjected to modulation; unphosphorylated cofilin is functionally active and cofilin phosphorylated at serine (S3) (p-cofilin) is functionally inactive. Retraction of filopodia/lamellipodia depends on contraction based on the interaction between F-actin and non-muscle myosin II (“myosin” from here onward) in which motor activity is triggered by myosin light chain kinase (MLCK) phosphorylating myosin light chain (MLC), which along with essential light chain (ELC) and myosin heavy chain (MHC) compose myosin (Clark et al., 2007). We showed in this context that efficient phagocytosis depended on Gal-3, enhancing the activation of both cofilin and MLCK (discussed in upcoming sections).

### Gal-3 promotes phagocytosis by enhancing K-Ras-dependent PI3K signaling in cultured primary microglia and macrophages

**Figure 2:**
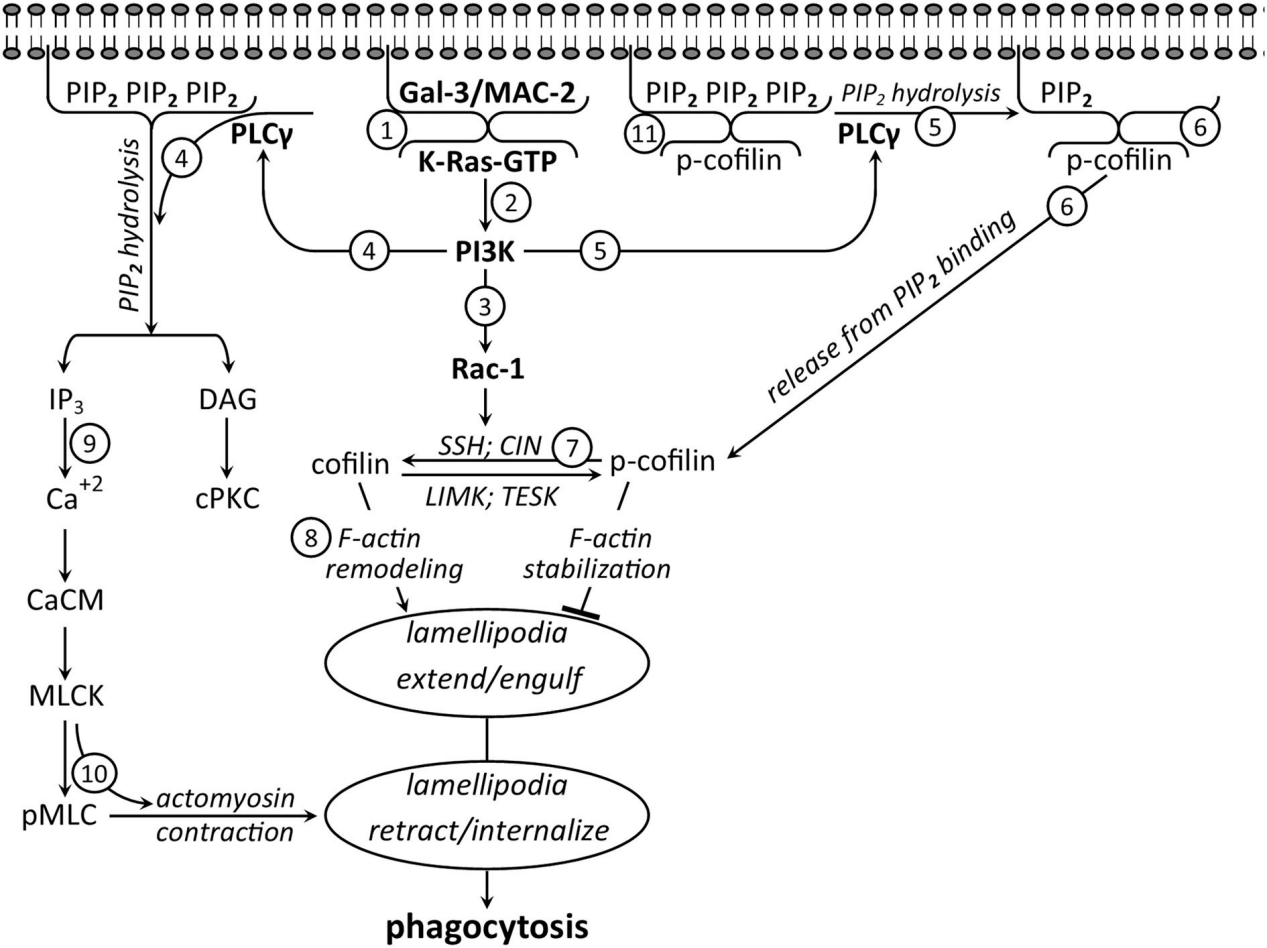
Intracellular Gal-3/MAC-2 takes center stage in promoting phagocytosis—a suggested signaling network based on the literature discussed in the text (not all signaling molecules that are involved in phagocytosis are shown). *Before phagocytosis*, cytoplasmic (1) Gal-3/MAC-2 and (11) PIP2 are attached to the inner surface of the plasma membrane, and further, (11) PIP2 binds inactive cofilin (p-cofilin), blocking its accessibility to activation by phosphatases. Phagocytosis starts as myelin debris (not shown) binds and activates CR3/MAC-1 (not shown) to generate the signaling network. Initially, (2) activated K-Ras-GTP forms a complex with Gal-3-MAC-2. In complex, (2) Gal-3/MAC-2 augments and prolongs K-Ras-GTP-dependent activation of PI3K, which, in turn, activates (3) Rac1 and (4 and 5) PLCγ. Consequently, (3) Rac1 activates phosphatases slingshot (SSH) and chronophin (CIN), (4) PLCγ hydrolyzes PIP2 into DAG and IP3, and (5) PLCγ-dependent hydrolysis of PIP2 (6) reduces PIP2 concentration at the plasma membrane, leading to the release of inactive p-cofilin from PIP2 inhibitory binding. Subsequently, (7) inactive p-cofilin becomes accessible to activation by phosphatases SSH and CIN that (3) Rac1 has activated. Then, (8) active cofilin advances F-actin remolding, leading to filopodia/lamellipodia extension and engulfment of myelin debris. Thereafter, (4) PIP2 hydrolysis product IP3 (9) increases Ca^+2^ concentration, leading to Ca^+2^/calmodulin (Ca^+2^/CaM)-dependent MLCK activation, followed by (10) MLCK phosphorylating MLC (MLC → pMLC), triggering motor activity in myosin. Motor-activated myosin interacts with F-actin to produce F-actin/myosin (actomyosin)-based contraction, leading to filopodia/lamellipodia retraction and debris internalization.

Two sets of observations suggest that intracellular Gal-3 promotes CR3/MAC-1- and SRAI/II-mediated myelin debris phagocytosis by enhancing the activation of K-Ras, a member of the RAS family of small GTPases. The first is our findings that phagocytosis depended on phosphatidylinositol 3-kinase (PI3K) activating phosphoinositide-specific phospholipase-Cγ (PLCγ), which, in turn, activated classical protein kinase-C (cPKC); i.e., PI3K → PLCγ → cPKC signaling cascade (Makranz et al., 2004; Cohen et al., 2006). The second is the findings by others in non-phagocytic cells (Elad-Sfadia et al., 2004; Plowman and Hancock, 2005; Ashery et al., 2006; Tian et al., 2010). First, K-Ras in its active state K-Ras-GTP activated PI3K. Second, Gal-3 preferentially formed a complex with K-Ras-GTP at the inner surface of plasma membranes. Third, Gal-3 in complex with K-Ras-GTP impeded the transition of active K-Ras-GTP to inactive K-Ras-GDP, thus augmenting and prolonging K-Ras-GTP-dependent PI3K signaling; i.e., K-Ras-GTP → PI3K signaling (Figure 2; step 2).

Together, the two sets of findings suggested that Gal-3 in complex with K-Ras-GTP promoted phagocytosis by augmenting and prolonging K-Ras-GTP → PI3K → PLCγ → cPKC signaling. Our subsequent studies and findings verified this proposition by showing the following (Rotshenker et al., 2008; Rotshenker, 2009). First, K-Ras-GTP levels and PI3K activity increased during normal phagocytosis. Second, blocking Gal-3 from forming a complex with K-RAS-GTP decreased K-Ras-GTP levels, PI3K activity, and phagocytosis. Third, Gal-3 co-immunoprecipitated with Ras, and the levels of the co-immunoprecipitate increased during normal phagocytosis and further decreased by blocking Gal-3 from forming a complex with K-RAS-GTP. These findings provide conclusive evidence that intracellular Gal-3 controls phagocytosis through an intracellular mechanism, yet without ruling out the Gal-3 ability to promote phagocytosis through an extracellular mechanism (discussed in upcoming sections).

### Gal-3 activates cofilin and phagocytosis and further induces amoeboid morphology in cultured primary microglia

**Figure 3:**
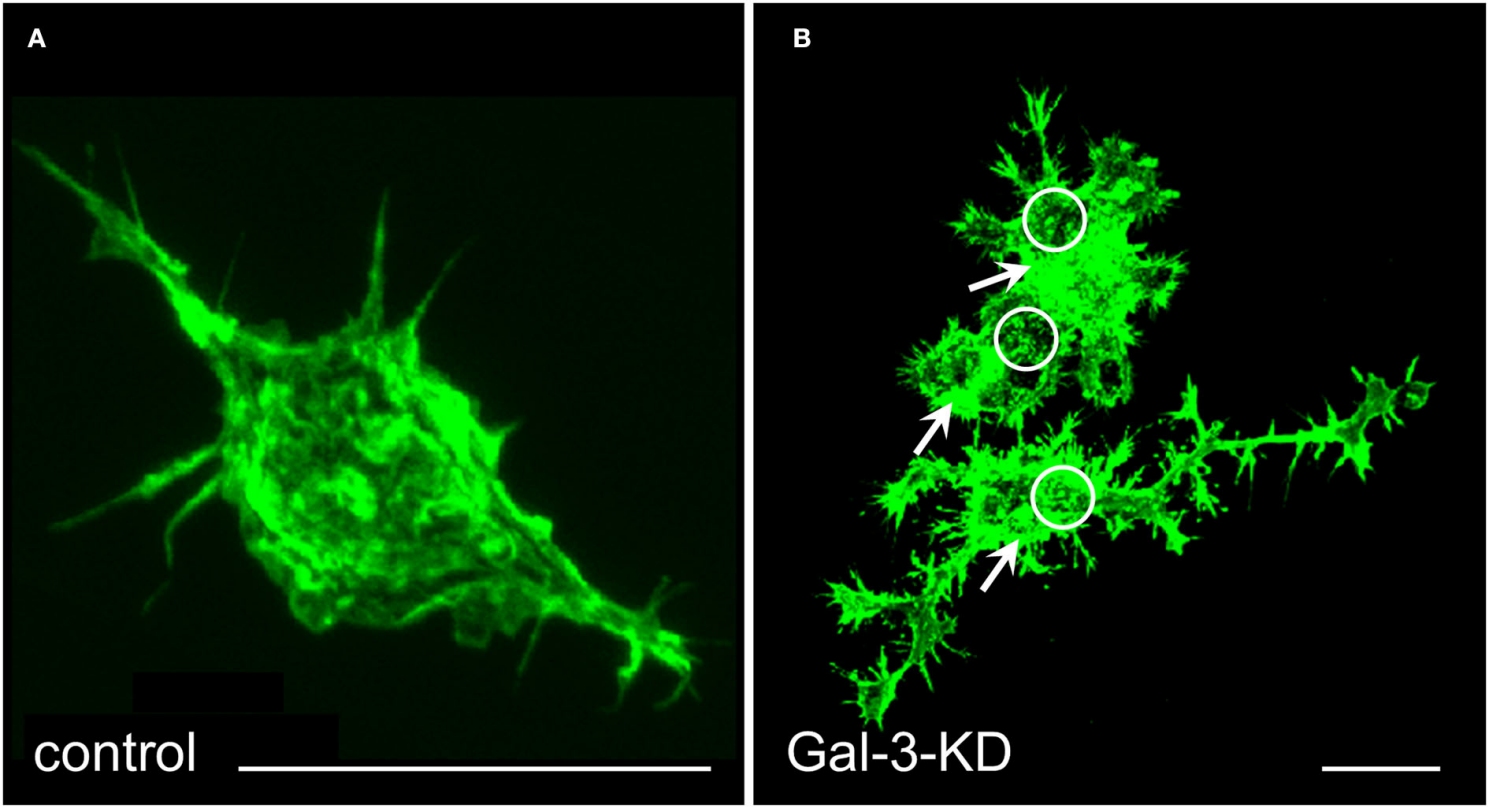
Gal-3/MAC-2 knocked-down (KD) microglia display branched-like morphology and organization of actin in dense punctate and massive deposits whereas control microglia display amoeboid-like morphology and organization of actin in filamentous structures. Immunofluorescence confocal microscopy images of **(A)** control microglia and **(B)** Gal-3-KD microglia; dense punctate and massive deposits of F-actin are respectively marked by circles and arrows. Alexa Fluor 488 labeled phalloidin (green) visualizes F-actin. Knocking down Gal-3 protein expression was achieved through lentiviral infection of wild-type microglia with shRNA directed against Gal-3/MAC-2 mRNA. As a control, wild-type microglia were infected in a similar way with the shRNA against the non-target firefly Luciferase gene. As seen, the image of control microglia in **(A)** is enlarged by a factor of 4 compared with the image of the Gal-3-KD microglia in **(B)**. Bars: 5 μm in **(A)** and 20 μm in **(B)**. Modified after Reichert and Rotshenker (2019).

We knocked down (KD) Gal-3 protein levels through lentiviral infection with shRNA against Gal-3 and further used as control microglia that were infected with the shRNA against the non-target firefly shRNA Luciferase gene (Reichert and Rotshenker, 2019). Gal-3-KD microglia displayed reduced levels of Gal-3 protein, deficient phagocytosis, and increased levels of inactive p-cofilin. Strikingly, knocking down Gal-3 resulted in a dramatic transformation of microglia morphology from “amoeboid-like” toward “branched-like” and rearrangement of actin filaments (Figure 3). These findings suggest that Gal-3 controlled microglia morphology and phagocytosis by regulating the activation state of cofilin, which, in turn, affected how actin filaments organize and how stable they are.

The inevitable question is, “How did Gal-3 promote cofilin activation and thereby phagocytosis?” Findings by others studying filopodia/lamellipodia extension and retraction in non-phagocytic cells and our findings studying myelin debris phagocytosis *via* CR3/MAC-1 and SRAI/II in primary microglia and macrophages together suggest that Gal-3 promoted cofilin activation through two signaling pathways downstream of PI3K. In the first pathway, PI3K-activated phosphatases slingshot (SSH) and chronophin (CIN) through small GTPase Rac1. In turn, SSH and CIN activated cofilin by dephosphorylating inactive p-cofilin (p-cofilin → cofilin) (Nishita et al., 2004; Raftopoulou and Hall, 2004; Kligys et al., 2007; Delorme-Walker et al., 2015) (Figure 2; steps 3 and 7).

In the second pathway, PI3K, through PLCγ, could increase the pool of inactive p-cofilin that is readily accessible to activation. It was shown (Oser and Condeelis, 2009; Bernstein and Bamburg, 2010; Senju and Lappalainen, 2019; Katan and Cockcroft, 2020) that inactive p-cofilin was not accessible to activation by SSH and CIN when bound to phosphatidylinositol (4,5) bisphosphate (PIP2) at the inner surface of the plasma membrane. However, p-cofilin became accessible to activation as PLCγ released p-cofilin from PIP2 inhibitory binding by reducing PIP2 concentration. PLCγ did so as it hydrolyzed PIP2 into inositol (1,4,5) trisphosphate (IP3) and diacylglycerol (DAG). Our findings suggest that this is most likely the case in phagocytosis too. We showed that Gal-3 in complex with K-Ras-GTP augmented and prolonged K-Ras-GTP → PI3K → PLCγ → cPKC signaling cascade, in which PLCγ-dependent PIP_2_ hydrolysis product, DAG, activated cPKC (Rotshenker et al., 2008; Rotshenker, 2009) (Figure 2; step 4). Since PLCγ hydrolyzed PIP_2_ in phagocytosis, reduced concentration of PIP_2_, leading to the release of inactive p-cofilin from PIP_2_ inhibitory binding, is highly likely (Figure 2; steps 5, 6, and 7).

Taken altogether, observations suggest that Gal-3 in complex with K-Ras-GTP augmented and prolonged K-Ras-GTP → PI3K signaling and thereby cofilin activation (p-cofilin → cofilin) in two complementary ways. First, it enhanced K-Ras-GTP → PI3K → Rac1-dependent activation of phosphatases SSH and CIN. Second, it augmented and prolonged K-Ras-GTP → PI3K → PLCγ-dependent hydrolysis of PIP_2_, resulting in an increased pool of inactive p-cofilin that is readily accessible to activation by phosphatases SSH and CIN (Figure 2; steps 5, 6 and 7).

### Gal-3 promotes phagocytosis by enhancing MLCK-dependent phosphorylation of MLC

**Figure 4:**
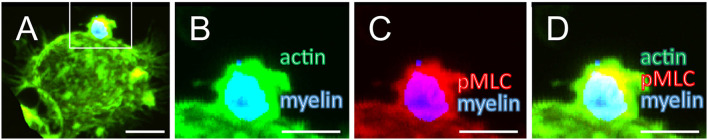
Myelin debris, F-actin, and pMLC colocalize at the phagocytic cup. Myelin (blue), pMLC (red), F-actin (actin; green), and overlap of actin and pMLC (yellow). **(A)** A phagocytic cup on top of microglia that phagocytoses myelin debris. **(B–D)** Enlargement of the phagocytic cup and its immediate surroundings [the area marked in **(A)**]. **(B)** Myelin and F-actin, **(C)** pMLC and myelin, **(D)** F-actin, pMLC and myelin. Optical slices, 1 μm thick, were scanned sequentially, and the entire phagocyte was reconstructed. Bars: 10 μm in **(A)** and 5 μm in **(B–D)**. Modified after Gitik et al. (2010).

We showed previously that internalization of myelin debris occurred through retraction of the filopodia/lamellipodia that had engulfed the debris (Hadas et al., 2012; Figure 1). The contractile forces that drive the retraction of filopodia/lamellipodia are generated through the interaction between F-actin and myosin, in which phosphorylation of MLC (MLC → pMLC) triggers motor activity. Two kinases, MLCK and Rho-kinase (ROCK), can phosphorylate MLC: MLCK at the periphery and ROCK at the center of cells (Totsukawa et al., 2000; Clark et al., 2007). We showed in this context that MLCK and not ROCK phosphorylated MLC at the phagocytic cup and, further, that inhibiting MLCK reduced both MLC phosphorylation and phagocytosis (Gitik et al., 2010; Figure 4); indicating that MLCK-dependent MLC phosphorylation promoted phagocytosis. Observations by others and by us together suggest that Gal-3 enhanced MLCK-dependent MLC phosphorylation. First, we showed that PI3K promoted phagocytosis through PLCγ-dependent PIP_2_ hydrolysis products IP_3_ and DAG (Makranz et al., 2004; Cohen et al., 2006). Second, others showed that IP_3_ increased Ca^+2^ levels (Berridge and Irvine, 1989), leading to the activation of calmodulin (CaM) that, in turn, activated MLCK (Crivici and Ikura, 1995; Raina et al., 2009). Third, we showed in this context that Ca^+2^ chelator BAPTA/AM inhibited phagocytosis (Makranz et al., 2004; Cohen et al., 2006). Thus, all findings together suggest that Gal-3 in complex with K-Ras-GTP enhanced MLC phosphorylation by enhancing K-Ras-GTP → PI3K → PLCγ → IP_3_ → Ca^+2^/CaM → MLCK → pMLC signaling cascade (Figure 2; steps 2, 4, 9 and 10).

In summary (Figure 2), intracellular Gal-3 takes center stage in promoting the phagocytosis of myelin debris *via* CR3/MAC-1 and SRAI/II. By forming a complex with K-Ras-GTP, Gal-3 augmented and prolonged K-Ras-GTP → PI3K-dependent signaling, targeting the cytoskeleton two time, first, by activating through Rac1 and PLCγ cofilin on which F-actin remodeling and engulfment of myelin debris depend, and second, by activating through PLCγ MLCK-dependent MLC phosphorylation on which F-actin/myosin based contraction and internalization of myelin debris depend. It is highly likely, though needing verification, that intracellular Gal-3 promotes phagocytosis *via* FcγR in a similar way (discussed below).

### Does intracellular Gal-3 promote FcγR-mediated phagocytosis of IgG-opsonized targets?

Extrapolating findings from one phagocytic system to another should be taken with great caution since signaling molecules may play different roles depending on the identity and nature of the phagocytic cell, receptor, and target (e.g., Gitik et al., 2010). Nonetheless, data from studies in which the role of signaling molecules depicted in Figure 2 were tested suggest that the signaling network proposed for myelin debris phagocytosis *via* CR3/MAC-1 and SRAI/II may also apply, at least in part, to phagocytosis of IgG-opsonized targets *via* FcγR.

### Gal-3

Sano et al. (2003) showed that deletion of Gal-3 from macrophages delayed phagocytosis of IgG-opsonized erythrocytes. This finding is in line with two observations regarding myelin debris phagocytosis *via* CR3/MAC-1 and SRAI/II. First, knocking down Gal-3 protein levels in microglia reduced the levels of both active cofilin and phagocytosis (Reichert and Rotshenker, 2019). Second, blocking intracellular Gal-3 from forming a complex with K-Ras-GTP reduced phagocytosis that K-Ras-GTP-dependent “eat me” signaling pathways promoted in microglia and macrophages (Rotshenker et al., 2008) (Figure 2; step 2).

### PI3K

Ninomiya et al. (1994) showed that ligation of FcγR activated PI3K and that PI3K promoted FcγR-mediated phagocytosis of IgG-opsonized erythrocytes in the human monocytic cell line U937 and Guinea pig neutrophils. In agreement, Araki et al. (1996) studying phagocytosis in bone-marrow-derived macrophages and Vieira et al. (2001) studying phagocytosis in RAW 264.7 cell line macrophages showed that PI3K was essential for FcγR-mediated phagocytosis. These findings are in line with our observations that PI3K promoted myelin debris phagocytosis *via* CR3/MAC-1 and SRAI/II in primary microglia and macrophages (Makranz et al., 2004; Cohen et al., 2006; Rotshenker et al., 2008; Rotshenker, 2009).

### PLCγ and PIP_2_

Botelho et al. (2000) and Scott et al. (2005) studied the roles of PLCγ and PIP_2_ in FcγR-mediated phagocytosis of IgG-opsonized erythrocytes in RAW 264.7 cell line macrophages. Together they showed and suggested the following: First, phagocytosis required the presence of active PLCγ and PIP_2_ at the phagosome, and the loss of PIP_2_ was correlated with PLCγ mobilization and with the localized formation of DAG. Second, PIP_2_ hydrolysis directed F-actin remodeling during phagocytosis, and inhibition of PIP_2_ hydrolysis prevented the actin disassembly necessary for phagocytosis. These findings are in line with our findings that PLCγ promoted phagocytosis of CR3/MAC-1- and SRAI/II-mediated myelin debris in primary macrophages and microglia through PIP_2_ hydrolysis products, DAG and IP_3_, that activated cPKC (Makranz et al., 2004; Cohen et al., 2006; Rotshenker, 2009). These findings are also in agreement with our suggestion that hydrolysis of PIP_2_ by PLCγ released p-cofilin from PIP_2_ inhibitory biding, increasing the pool of inactive p-cofilin that was readily accessible to activation, leading to increased levels of active cofilin that initiated F-actin remodeling and engulfment of phagocytic targets (discussed above and Figure 2).

### Rac1

Studying FcγR-mediated phagocytosis of IgG-opsonized erythrocytes and latex beads in RAW 264.7 cell line macrophages, Ikeda et al. (2017) showed that Rac1 activation was essential at the early stages of phagocytosis as lamellipodia extended and engulfed targets. This finding is in line with the following observations. First, PI3K activated phosphatases SSH and CIN through small GTPase Rac1, directing SSH and CIN to activate cofilin by dephosphorylating inactive p-cofilin (p-cofilin → cofilin) (Nishita et al., 2004; Raftopoulou and Hall, 2004; Kligys et al., 2007; Delorme-Walker et al., 2015). Second, our findings that active cofilin, on which F-actin remodeling and target engulfment depended, promoted phagocytosis (Hadas et al., 2012; Gitik et al., 2014; Reichert and Rotshenker, 2019) (Figure 2; steps 3, 5, 6, 7, and 8).

Of note, in their model, Ikeda et al. (2017) assigned Rac1 a role in F-actin assembly by actin polymerization. Herein, the role assigned to Rac1 is to advance SSH- and CIN-dependent activation of cofilin, leading to F-actin disassembly (discussed above and Figure 2). The two roles, disassembly and assembly, complement each other since lamellipodia extension occurs through F-actin remodeling. Initially, existing F-actin disassembles through depolymerization and then new F-actin reassembles in a new configuration through actin polymerization.

### pMLC

Ikeda et al. (2017) further showed that myosin and pMLC colocalized at the phagosome, indicating that motor activity had been triggered in myosin. This finding is in line with our findings that pMLC, F-actin, and myelin debris colocalized at the phagocytic cup and that phosphorylation of MLC promoted phagocytosis (Gitik et al., 2010) (Figure 4).

*In conclusion*, it is highly likely that Gal-3, PI3K, PLCγ, PIP_2_, Rac1, and pMLC play similar roles in promoting the phagocytosis of IgG-opsonized targets *via* FcγR as in promoting the phagocytosis of myelin debris *via* CR3/MAC-1 and SRAI/II (Figure 2).

## Extracellular Gal-3 promotes phagocytosis by functioning as an opsonin

Extracellular Gal-3 can promote phagocytosis. This was first suggested by findings that exogenously applied lactose and galactose that block Gal-3 binding β-galactoside reduced phagocytosis of myelin debris in Schwann cells (Reichert et al., 1994) and that exogenously applied Gal-3 augmented lactose-inhibitable phagocytosis of apoptotic cells in macrophages (Karlsson et al., 2009). However, these findings did not disclose the underlying mechanism in detail.

### Macrophages

Caberoy et al. (2012) provided evidence that extracellular Gal-3 can function as an opsonin. Studying the phagocytosis of apoptotic cells in cultured J774 macrophages, they showed that Gal-3 linked phagocytic receptor MerTK and targets (apoptotic cells and tissue debris), leading to MerTK activation, autophosphorylation, and phagocytosis. Co-immunoprecipitation of MerTK with Gal-3 and blocking phagocytosis by exogenously applying soluble MerTK extracellular domain and lactose further verified that Gal-3 ligated and activated MerTK-mediated phagocytosis. Notably, MerTK ligands Gas6 and Protein S function as opsonins in MerTK-dependent phagocytosis of apoptotic cells too (Lemke, 2013).

### Microglia

Studying phagocytosis of stressed, dead and live neurons, and bacteria in cultured BV-2 cell lines and primary microglia, Nomura et al. (2017) and Cockram et al. (2019) provided evidence that Gal-3 binds both MerTK and targets and that lactose and MerTK inhibitor inhibited phagocytosis. Notably, phagocytosis of stressed, dead, and live neurons depended on the prior treatment of the microglia with lipopolysaccharide (LPS) that increased MerTK expression and Gal-3 secretion, suggesting that the levels of MerTK expression and/or Gal-3 secretion were too low in untreated microglia to carry out MerTK-mediated phagocytosis of Gal-3-opsonized targets. Moreover, sialic acid often binds galactose residues on cell surface phagocytic receptors (e.g., MerTK) and phagocytic targets as does Gal-3, thus impeding phagocytosis by blocking Gal-3 binding/crosslinking MerTK and targets (reviewed in Puigdellivol et al., 2020). Nomura et al. (2017) and Allendorf and Brown (2022) showed in this context that the LPS-treated microglia increased the release of Neuraminidase 1 (Neu1), which through desialylation exposed galactose residues on MerTK and targets, thus leading to augmented phagocytosis by enabling Gal-3 binding and so crosslinking MerTK and targets. Taken altogether, efficient MerTK-mediated phagocytosis of Gal-3-opsonized targets depended on inflammatory stimuli activating microglia to upregulate MerTK expression and further increase Gal-3 and neuraminidase secretion.

### Schwann cells

Following PNS nerve injury, axons and myelin break in PNS Wallerian degeneration and then resident Schwann cells and recruited macrophages clear the debris (e.g., Rotshenker, 2011, 2015). That recruited macrophages clear debris by phagocytosis has long been accepted, whereas phagocytosis by Schwann cells was debated even in the face of findings by Bigbee et al. (1987) that cultured Schwann cells internalized exogenously applied myelin debris. Reichert et al. (1994) showed that Schwann cells in cultured PNS nerve explants undergoing Wallerian degeneration *in vitro* in the absence of recruited macrophages expressed Gal-3 (named MAC-2 in this study) and cleared myelin debris, which exogenously applied lactose and galactose inhibited suggesting that extracellular Gal-3 promoted debris phagocytosis in Schwann cells. Brosius et al. (2017) showed that Schwann cells in PNS Wallerian degeneration upregulated MerTK and Axl expression and, further, that MerTK and Axl mediated myelin debris phagocytosis. The following findings together suggest that Gal-3 could function as an opsonin in MerTK-mediated phagocytosis of myelin debris in Schwann cells. First, Schwann cells produced Gal-3 and internalized myelin debris *in vitro* in culture and *in vivo* in PNS Wallerian degeneration (Bigbee et al., 1987; Reichert et al., 1994). Second, MerTK mediated myelin debris phagocytosis in Schwann cells (Brosius et al., 2017). Third, Gal-3 ligated MerTK (Brosius et al., 2017) and myelin (Probstmeier et al., 1995). Fourth, lactose and galactose inhibited myelin debris phagocytosis in Schwann cells (Reichert et al., 1994). Notably, Schwann cells in PNS Wallerian degeneration internalize disrupted myelin also by autophagy (Gomez-Sanchez et al., 2015; Brosius et al., 2017).

In intact peripheral nerves, Schwann cells produce myelin sheaths that surround motor axons. By contrast, Schwann cells at the neuromuscular junction/synapse do not produce myelin; they cover the motor axon endings, the presynaptic elements of the synapse, but avoid contact with the nerve-ending surface facing the muscle fiber. Martineau et al. (2020) studied, among others, the clearance of degenerating axonal synaptic endings in wild-type mice and *SOD1*^*G*37*R*^ mice, an animal model for amyotrophic lateral sclerosis. They showed that synaptic Schwann cells produced and secreted Gal-3 and phagocytosed degenerating axonal nerve endings following PNS nerve injury in wild-type mice. Thus, synaptic Schwann cells at the degenerating neuromuscular synapse that do not produce myelin mimicked Schwann cells in PNS Wallerian degeneration that normally produce myelin: both phagocytosed debris and both expressed Gal-3. Therefore, it is conceivable but needs verification that Gal-3 functioned as an opsonin to promote phagocytosis of disrupted nerve endings *via* MerTK in synaptic Schwann cells as it promoted phagocytosis of myelin debris *via* MerTK in Schwann cells in PNS Wallerian degeneration (discussed above).

### Astrocytes

Nguyen et al. (2011) showed that optic nerve axons at the myelination transition zone normally protruded vesicular-like structures that normal Gal-3 expressing astrocytes phagocytosed. Chung et al. (2013) showed that MerTK-expressing astrocytes contributed to developmental synaptic pruning/elimination in the lateral geniculate body *via* MerTK. Although not tested directly, the findings that astrocytes that phagocytosed axonal elements expressed Gal-3 (Nguyen et al., 2011) and MerTK (Chung et al., 2013) suggest the possibility that Gal-3 functioned as an opsonin in MerTK-mediated phagocytosis of axonal elements in astrocytes. Notably, both microglia and astrocytes carry out developmental synapse pruning/elimination in the developing CNS, microglia *via* CR3/MAC-1, and astrocytes *via* MerTK and MEGF10 (Schafer et al., 2012; Chung et al., 2013; Faust et al., 2021).

*In conclusion*, extracellular Gal-3 promotes phagocytosis in professional phagocytes (microglia and macrophages) *via* MerTK by functioning as an opsonin. It is highly likely, though needs final verification, that extracellular Gal-3, functioning as an opsonin, promotes phagocytosis *via* MerTK also in non-professional phagocytes (Schwann cells and astrocytes).

## Gal-3 controls macropinocytosis in some types of cancer cells

Macropinocytosis is an evolutionary conserved non-selective endocytic mechanism that various cell types (e.g., malignant cells and macrophages) use for the uptake of extracellular fluid with included dissolved nutrients (e.g., amino acids and glucose), proteins (e.g., albumin and antigens), pathogens (e.g., bacteria and viruses), and debris; e.g., (King and Kay, 2019; Palm, 2019; Kay, 2021). Macropinocytosis and phagocytosis depend on similar structural changes that the cytoskeleton drives; e.g., (Mylvaganam et al., 2021). In macropinocytosis, plasma membrane ruffles/lamellipodia engulf the bulk of extracellular fluid and included materials/cargo. Then, ruffles/lamellipodia internalize the fluid and cargo into vesicular-shaped macropinosomes that range in a diameter of 0.2 to 5 μm. Whereas phagocytosis activation depends on phagocytic targets/cargo ligating cognate phagocytic receptors, the activation of macropinocytosis does not. Macropinocytosis may be triggered by growth factors (e.g., epidermal growth factor) and Ca^+2^ or occur autonomously (Canton, 2022; Lambies and Commisso, 2022; Palm, 2022). Nonetheless, macropinocytosis and phagocytosis share many signaling molecules that are involved in directing the cytoskeleton to produce the mechanical forces that drive the structural changes on which they depend; e.g., K-Ras, PI3K, Rac, PLC, PIP_3_ and PIP_2_, and PKC; (Swanson, 2008; Zhang and Commisso, 2019; Lambies and Commisso, 2022; Palm, 2022).

Studying lung cancer cells, Seguin et al. (2017) identified a subset of lung cancer cells that expressed an oncogenic K-Ras mutant and high levels of both Gal-3 and integrin αvβ3. The survival and progression of these cancer cells relied on Gal-3-dependent macropinocytosis. The authors showed that binding of extracellular Gal-3 to αvβ3 integrin resulted in αvβ3 clustering, leading to K-Ras signaling and macropinocytosis and, further, to reduced production of reactive oxygen species (ROS). By contrast, blocking gal-3 binding to αvβ3 and reducing Gal-3 expression each suppressed macropinocytosis and further increased ROS production and cell death.

Studying glioblastoma patient-derived stem cells (GSCs), Seguin et al. (2021) characterized a subset of glioblastoma (GBM) tumors that expressed non-oncogenic K-Ras and high levels of Gal-3. The GSCs relied on Gal-3-dependent macropinocytosis for their survival as knocking down Gal-3 resulted in reduced macropinocytosis, survival, and invasion of GSCs. The authors further showed the following. First, extracellular Gal-3 interacted with β1 integrin. Second, intracellular Gal-3 interacted with small GTPase RAB10 that is involved in macropinosome formation and maturation (see below). Third, knocking down either RAB10 or β1 integrin reduced macropinocytosis. Altogether, these findings suggested to the authors that both extracellular Gal-3 binding/clustering β1 integrin and intracellular Gal-3 binding RAB10 activated macropinocytosis in GSCs.

### Does Gal-3 control macropinocytosis in macrophages?

Studying, among others, primary bone-marrow-derived macrophages and RAW 364.7 macrophages, Liu et al. (2020) showed that knocking-down small GTPase Rab10 expression reduced macropinocytosis without affecting phagocytosis or clathrin-mediated endocytosis and that Rab10 was recruited to PIP_2_/PIP_3_ rich membrane ruffles at the early stage of macropinosome formation. These findings suggest that small GTPase Rab10 controls macropinocytosis by regulating the formation and maturation of macropinosomes. Taken that (a) Gal-3 promotes RAB10-dependent macropinocytosis in GBM stem cells (discussed above) and (b) macrophages produce Gal-3 under inflammatory conditions, it is highly likely, but needs verification, that Gal-3 promotes RAB10-dependent macropinocytosis in macrophages too.

## Gal-3 controls lysosomal repair and lysophagy

Macropinosomes formed in macropinocytosis and phagosomes in phagocytosis fuse with lysosomes to degrade internalized targets/cargo. Some targets/cargo (e.g., viruses, bacteria, and neurotoxic aggregates) can damage/rupture lysosomal membranes, resulting, among others, in cell death and inflammation unless lysosomal membranes undergo either repair or removal (e.g., recently reviewed in Jia et al., 2020; Papadopoulos et al., 2020; Zhu et al., 2020; Hong et al., 2021). Gal-3 plays a central role in repair and removal. As lysosomal membranes (and other endo-membranes) rupture, cytosolic galectins gain access to glycosylated proteins on the inner surface of the endo-membranes. Studying repair and removal of damaged lysosomes, Jia et al. (2020) showed and further suggested that, following lysosomal damage, Gal-3 recruited protein ALIX, leading to repair and restoration of lysosomal function. Thereafter, if repair failed, Gal-3 recruited tripartite motif (TRIM) protein TRIM16, leading to lysophagy. Notably, drug-induced lysosomal damage and inhibition of lysophagy have been suggested as a potential therapeutic strategy in treating GBM (Jing et al., 2022).

## Concluding remarks

Gal-3 controls phagocytosis in “professional phagocytes” (e.g., macrophages and microglia) and “non-professional phagocytes” (e.g., Schwann cells and astrocytes) either through intracellular and/or extracellular mechanisms, intracellularly by controlling the “eat me” signaling pathways or extracellularly by functioning as an opsonin. Each of these cells produces and secretes Gal-3 under either normal conditions and/or pathological conditions, indicating that production and secretion are subject to regulation. Mechanisms that control Gal-3 expression, production, and secretion remain largely unknown.

Conclusive evidence indicates that intracellular Gal-3 promotes myelin debris phagocytosis *via* CR3/MAC-1 in microglia and macrophages by controlling the “eat me” signaling pathways. The current knowledge suggests that it is highly likely that intracellular Gal-3 promotes phagocytosis *via* FcγR in macrophages in a similar way. It is yet unknown, and thus needs testing, if intracellular Gal-3 controls phagocytosis *via* additional phagocytic receptors (e.g., MerTK). In development, microglia that normally express Gal-3 prune/strip/eliminate synapses by phagocytosis *via* CR3/MAC-1. Whether intracellular Gal-3 controls developmental synaptic pruning in microglia is likely but needs confirmation.

Conclusive evidence indicates that Gal-3 promotes phagocytosis of neurons, apoptotic cells, bacteria, and debris *via* MerTK in macrophages and microglia by functioning as an opsonin. Current knowledge suggests that it is highly likely that Gal-3 promotes phagocytosis of myelin debris and axonal synaptic nerve endings *via* MerTK in Schwann cells and astrocytes in a similar way.

Notably, Gal-3 promotes phagocytosis in microglia and macrophages through both intracellular and extracellular mechanisms, intracellularly by advancing the “eat me” signaling pathways in phagocytosis and extracellularly by functioning as an opsonin. Whether Gal-3 promotes phagocytosis in Schwann cells and astrocytes by both intracellular and extracellular mechanisms remains to be determined. The current knowledge reveals that Gal-3 promotes phagocytosis in microglia and macrophages *via* different phagocytic receptors, intracellularly *via* CR3/MAC-1 and FcγR and extracellularly *via* MerTK. Whether Gal-3 promotes phagocytosis intracellularly and extracellular *via* the same phagocytic receptor (e.g., MerTK) remains to be determined.

Gal-3 controls macropinocytosis in some types of cancer through extracellular and intracellular mechanisms. The current knowledge suggests that these mechanisms are distinct from those involved in phagocytosis. In macropinocytosis, extracellular Gal-3 binding/clustering integrins (e.g., β1 and αvβ3) lead to K-Ras signaling and macropinocytosis. In phagocytosis, extracellular Gal-3 functions as an opsonin linking targets with cognate phagocytic receptors. Intracellular Gal-3 controls macropinocytosis by regulating macropinosome formation through interaction with small GTPase RAB10. In phagocytosis, intracellular Gal-3 controls phagocytosis through interaction with the small GTPase K-Ras. It remains unclear, and thus needs testing, if and under what conditions (e.g., inflammation) Gal-3 controls macropinocytosis in other cell types (e.g., macrophages and microglia).

## Author contributions

SR wrote the manuscript and headed the original cited work by SR and his research teams.

## Funding

Original cited work by SR and his research teams was supported by grants from the Bernard Schoenfeld family, the Yeshaya Horowitz Association, Sir Cowen Hebrew University funds, the US–Israel Binational Science Foundation, the Israel Science Foundation, the Niedersachsen-Israeli Research Cooperation fund, and the Charles Wolfson Charitable Trust.

## Conflict of interest

The author declares that the research was conducted in the absence of any commercial or financial relationships that could be construed as a potential conflict of interest.

## Publisher's note

All claims expressed in this article are solely those of the authors and do not necessarily represent those of their affiliated organizations, or those of the publisher, the editors and the reviewers. Any product that may be evaluated in this article, or claim that may be made by its manufacturer, is not guaranteed or endorsed by the publisher.
